# Two-Round Treatment With Propidium Monoazide Completely Inhibits the Detection of Dead *Campylobacter* spp. Cells by Quantitative PCR

**DOI:** 10.3389/fmicb.2022.801961

**Published:** 2022-04-25

**Authors:** Ayaka Okada, Mizuki Tsuchida, Md. Matiur Rahman, Yasuo Inoshima

**Affiliations:** ^1^Laboratory of Food and Environmental Hygiene, Faculty of Applied Biological Sciences, Cooperative Department of Veterinary Medicine, Gifu University, Gifu, Japan; ^2^Education and Research Center for Food Animal Health, Gifu University (GeFAH), Gifu, Japan; ^3^The United Graduate School of Veterinary Sciences, Gifu University, Gifu, Japan; ^4^Department of Medicine, Faculty of Veterinary, Animal and Biomedical Sciences, Sylhet Agricultural University, Sylhet, Bangladesh; ^5^Joint Graduate School of Veterinary Sciences, Gifu University, Gifu, Japan

**Keywords:** *Campylobacter*, culture method, propidium monoazide, quantitative PCR, viable but non-culturable

## Abstract

*Campylobacter* spp. are known as important foodborne gastroenteric pathogens worldwide. *Campylobacter* spp. can exist in a viable but non-culturable (VBNC) state under unsuitable environmental conditions, which is undetectable by conventional culture methods. Quantitative polymerase chain reaction (qPCR) can be used to detect VBNC *Campylobacter* spp.; however, both viable and dead bacteria are detected during qPCR and are indistinguishable. Propidium monoazide (PMA), which can only enter dead bacterial cells through a damaged cell wall/cell membrane, binds to DNA and inhibits qPCR. PMA treatment has been performed along with qPCR (PMA-qPCR) to detect viable bacteria. However, the efficacy of detection inhibition differed among studies, and PMA can potentially enter living cells after changes in cell membrane permeability. In this study, we optimized the PMA treatment method by conducting it before qPCR. Two-round PMA treatment completely inhibited the qPCR signals from dead cells, whereas single-round PMA treatment failed to facilitate this. An optimized PMA-qPCR method was developed using commercial chicken meat, and VBNC *Campylobacter* spp., which are undetectable using conventional culture-based methods, were successfully detected. In conclusion, this study presents a novel, efficient PMA treatment method for the detection of viable *Campylobacter* spp., including VBNC *Campylobacter* spp., in chicken meat. We believe that this method will aid the reliable risk assessment of commercial chicken meat.

## Introduction

Campylobacteriosis is one of the most common causes of foodborne bacterial diarrhea worldwide and is mostly associated with poultry meat (Skirrow, 1977; Humphrey et al., 2007). According to the Food Poisoning Statistics of the Ministry of Health, Labour, and Welfare in Japan, *Campylobacter jejuni* and *C. coli* are the most common bacteria implicated in incidents of food poisoning reported since 2003 in the country (Vetchapitak and Misawa, 2019).

The prevalence of *Campylobacter* spp. in poultry has been monitored most commonly using conventional culture-based methods. However, *Campylobacter* is known to exist in a viable but non-culturable (VBNC) state under various environmental stresses, such as low temperature, nutrient starvation, and unsuitable aerobic conditions (Hazeleger et al., 1995; Jackson et al., 2009; Oh et al., 2015). Culture-based methods cannot detect bacteria in the VBNC state; thus, polymerase chain reaction (PCR) or quantitative PCR (qPCR) should be performed to detect viable bacteria in samples. However, PCR or qPCR amplifies DNA from both viable and dead bacteria. To address this issue, propidium monoazide (PMA) treatment combined with PCR or qPCR has been performed in several studies for detecting viable bacteria (Zeng et al., 2016). PMA only enters dead bacteria through the damaged cell wall/cell membrane, binds to the DNA, and prevents DNA amplification *via* PCR (Nocker et al., 2006). Although ethidium monoazide has also been used to inhibit the detection of dead bacteria *via* the same mechanism of action as PMA, ethidium monoazide partially penetrates viable bacterial cells (Nocker et al., 2006). Because the number of *Campylobacter* spp. cells naturally contaminating poultry meat is relatively small (Scherer et al., 2006; Duqué et al., 2018; Ohnishi and Hara-Kudo, 2021), the detection of viable bacteria using PMA may be a more suitable option, as shown previously (Josefsen et al., 2010; Pacholewicz et al., 2013; Duarte et al., 2015; Lv et al., 2019).

Josefsen et al. (2010) reported the detection of viable *Campylobacter* spp. in chicken carcasses using PMA treatment combined with qPCR (PMA-qPCR); however, recent studies have shown that PMA-qPCR does not fully prevent the detection of dead cells (Pacholewicz et al., 2013; Seinige et al., 2014; Duarte et al., 2015). Lv et al. (2019) reported that PMA-qPCR with the intercalating dye SYBR Green could help detect viable *Campylobacter* cells among dead cells present at a concentration of 6 log colony-forming units (CFUs)/mL. As observed for other pathogens, TaqMan probe-based qPCR showed higher specificity than SYBR Green-based qPCR in the detection of *Campylobacter* spp. (Botteldoorn et al., 2008). Therefore, in this study, we performed TaqMan probe-based qPCR in the PMA-qPCR experiment.

PMAxx, a modified version of PMA, has recently been developed by Biotium, Inc. (Fremont, CA, United States) to improve the reactivity of PMA. In addition, the commercial product PMA Enhancer for Gram-negative bacteria (Biotium, Inc.), which improves the affinity of PMA to DNA from dead cells, can be combined with PMA to improve the selective detection of viable cells. However, studies performed using *Legionella pneumophila* and *Xylella fastidiosa* showed that qPCR signals from both viable and dead cells were inhibited when PMA Enhancer was used in combination with PMA, and the authors recommended the use of PMA (or PMAxx) without PMA Enhancer (Kontchou and Nocker, 2019; Sicard et al., 2019).

This study aimed to optimize PMA treatment for distinguishing viable cells from dead cells to facilitate the reliable detection of viable *Campylobacter* spp., including VBNC cells. We assessed the suitability of PMA Enhancer and the optimal concentration of PMAxx for TaqMan probe-based qPCR. In addition, we compared the efficiency of the optimized PMA-qPCR method with that of a conventional culture-based method for the detection of *Campylobacter* spp. in chicken meat.

## Materials and Methods

### Bacterial Strains and Culture Conditions

The *C. jejuni* strain JCM 2013 was obtained from the Japan Collection of Microorganisms (Tsukuba, Japan). Cells from the stock maintained at −80°C were cultured for 2 days on modified charcoal-cefoperazone-deoxycholate agar (mCCDA) [*Campylobacter* blood-free selective medium (Oxoid, Hampshire, UK) with CCDA selective supplement (Oxoid)] at 37°C under microaerobic conditions. In all experiments performed for this study, we established microaerobic conditions using the AnaeroPack-MicroAero agent (Mitsubishi Gas Chemical, Tokyo, Japan) in an anaerobic jar (Mitsubishi Gas Chemical). The bacteria were picked from mCCDA, suspended in 10 ml of Brucella broth (BD Biosciences, San Jose, CA, United States), and cultured for 36 h at 37°C under microaerobic conditions with shaking at 130 rpm using an incubator shaker (Innova 4300, New Brunswick Scientific, Enfield, CT, United States). The optical density of the liquid cultures at 600 nm was adjusted to 0.5 using a spectrophotometer (GeneQuant 100, GE Healthcare, Chicago, IL, United States), which corresponded to a concentration of approximately 10^8^ CFU/ml. All bacterial cells used in this study were prepared as follows: bacterial suspensions of various concentrations were prepared by serial dilution of the 10^8^ CFU/ml solution and were used as the source of viable cells. Dead cells were generated by heating the suspensions at 95°C for 5 min as described previously (Josefsen et al., 2010) in a thermoblock (ND-MD1, NISSIN, Tokyo, Japan). Cell viability was examined using a LIVE/DEAD BacLight Bacterial Viability Kit (Invitrogen, Carlsbad, CA, United States). The sample solutions were stained by a mixture (1:1) of SYTO9 and propidium iodide provided in the kit. The stained cells were mounted on glass slides and examined using an epifluorescence microscope (ECLIPSE 80i, Nikon, Tokyo, Japan). Viable cells were estimated by counting cells with green fluorescence, without red fluorescence, and dead cells were estimated by counting cells with red fluorescence. Cell counting was conducted in four random fields with an area of 112.5 μM × 90 μM on the filter at 400× magnification for each sample. Bacterial viability (%) was defined as the ratio of viable cells to total cells.

### PMA Treatment and DNA Extraction

To 160 μl of the samples containing cells at various concentrations, 40 μl of PMA Enhancer for Gram-negative bacteria (5× solution; Biotium, Inc., Fremont, CA, United States) or Brucella broth (for the negative control) was added. PMAxx solution (20 mM in H_2_O; Biotium, Inc.) was added to 200 μl of the samples to achieve final concentrations of 25, 50, and 100 μM. The samples were mixed and incubated for 10 min in the dark at 37°C, and then exposed to light for 15 min using an LED Crosslinker 12 (Takara, Kusatsu, Japan) to activate PMAxx. To investigate the effects of single-round PMA treatment, DNA was extracted from the PMA-treated cells without centrifugation. For investigating the effects of second-round PMA treatment, the cells were centrifuged at 8,000 × *g* for 10 min at 4°C, and the pellets were resuspended in 160 μl of phosphate-buffered saline (PBS) and treated with PMA Enhancer and 25 μM PMAxx, as described above. DNA was extracted using a DNeasy Blood & Tissue Kit (Qiagen, Hilden, Germany) according to the manufacturer’s instructions. The elution volume was 100 μl.

### Enumeration Using qPCR

The extracted DNA was used for qPCR analysis performed using a StepOnePlus thermal cycler (Applied Biosystems, Foster City, CA, United States). Specific primers and a probe were used to evaluate the presence of *Campylobacter* spp. (Table 1). The ΔCt of a sample is the difference between the Ct value obtained with PMA-treated sample and the Ct value obtained with an untreated sample. The PCR mixture was prepared to a final volume of 20 μl, which contained 2 μl of the extracted DNA, using the GoTaq Probe qPCR Master Mix (Promega, Madison, WI, United States) according to the manufacturer’s instructions. Thermal cycling was performed as follows: 2 min at 95°C, followed by 40 cycles of 3 s at 95°C and 30 s at 60°C. Fluorescence intensity was measured at the end of each cycle.

**Table 1:** Primers and probes used in this study.

Primer or Probe	Nucleotide sequence (5′-3′)	Gene
CampF2	CACGTGCTACAATGGCATAT	16S rRNA
CampR2	GGCTTCATGCTCTCGAGTT	
CampP2	FAM-CAGAGAACAATCCGAACTGGGACA- BHQ1	
C412F	GGATGACACTTTTCGGAGC	16S rRNA
C1228R	CATTGTAGCACGTGTGTC	

### Preparation of Chicken Juice

Twenty grams of chicken meat uncontaminated by *Campylobacter* spp. was mixed with 20 ml of PBS in a sterile stomacher bag (Kanto Kagaku, Tokyo, Japan) and homogenized for 1 min using a stomacher (BAGMIXER 400, Interscience, St. Norm, France). The chicken juice was collected through a filter in the stomacher bag and used for further experiments.

### Chicken Meat

A total of 26 chicken meat packs were purchased from three local supermarkets in Gifu Prefecture, Japan, in September 2021. Nine whole legs, nine breast fillets, and eight livers were used from the purchased meat. The samples were transported to the laboratory in isothermal boxes and isolated within 1 h of purchase.

For PMA-qPCR, 25 g of the chicken samples were rinsed with 10 ml of PBS. The rinse solution was centrifuged at 800 × *g* for 10 min at 4°C to eliminate larger debris, and the supernatant was centrifuged at 8,000 × *g* for 10 min at 4°C to precipitate *Campylobacter* spp. The pellet was resuspended in 160 μl of PBS and used for the two-round PMA treatment, as described above.

### Culture-Based Enumeration

*Campylobacter* spp. were detected according to the NIHSJ-02 guideline, which reports the standard detection method for *Campylobacter* spp. in Japan (Momose et al., 2013). Approximately 25 g of each sample was mixed with 100 ml of Preston selective broth [nutrient broth No.2 (Oxoid) base with 5% sterile lysed horse blood, *Campylobacter* growth supplement (Oxoid), and modified Preston *Campylobacter* selective supplement (Oxoid)]. The samples were mixed for 1 min using a stomacher and then incubated for 24 h at 42°C under microaerobic conditions. Enrichment cultures were streaked on mCCDA and Skirrow agar [Blood agar base No.2 (Oxoid) with 7% sterile lysed horse blood, Skirrow *Campylobacter* selective supplement (Oxoid), and *Campylobacter* growth supplement]. The cultures were incubated for 48 h at 42°C under microaerobic conditions. A presumptive *Campylobacter* colony was selected from each sample and identified by PCR of colony materials using the C412F and C1228R primers (Linton et al., 1996; Table 1). The amplicons were purified by ethanol precipitation and sequenced with the C412F or C1228R primers in an ABI 310 Genetic Analyzer using the Big Dye Terminator v3.1 Cycle Sequencing Kit (Applied Biosystems). The obtained sequences were analyzed using the NCBI BLAST search program.

### Statistical Analysis

All experiments were conducted with at least three independent replicates. Data were analyzed for statistical significance using Tukey’s post-hoc test.

## Results

In all experiments, the viability of viable cells was confirmed to be more than 90% and that of dead cells was confirmed to be 0% using the LIVE/DEAD BacLight Bacterial Viability Kit.

### The Effect of PMA Enhancer

**Figure 1:**
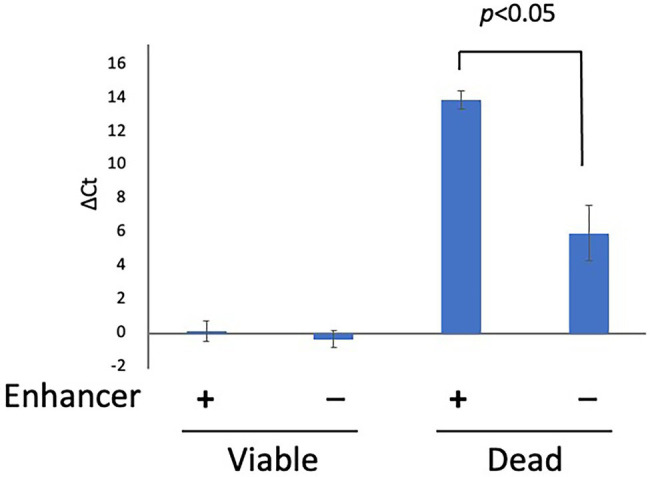
The effect of PMA Enhancer. Two hundred microliters each of viable and dead cell suspensions (concentration: 5 × 10^7^ CFU/ml for both) was treated with PMA Enhancer or Brucella broth, and 25 μM PMAxx was added prior to DNA extraction and qPCR. The columns display mean values from three independent experiments ± standard deviation. PMA, propidium monoazide; CFU, colony-forming unit; and qPCR, quantitative PCR.

To examine the inhibitory effect of PMA Enhancer on the qPCR signals from dead cells, 200 μl each of the viable cell and dead cell suspensions (concentration: 5 × 10^7^ CFU/ml for both) was prepared as described in “Bacterial Strains and Culture Conditions”, treated with PMA Enhancer or Brucella broth, and 25 μM PMAxx was added before DNA extraction and qPCR. In the viable cell suspension, there were no differences between the qPCR signals from the cells treated with the PMA Enhancer and those treated with the Brucella broth; however, in the dead cell suspension, the addition of PMA Enhancer significantly inhibited the qPCR signals (Figure 1). This result indicated that PMA Enhancer is useful for distinguishing viable cells from dead cells.

### Optimization of PMA Treatment

**Figure 2:**
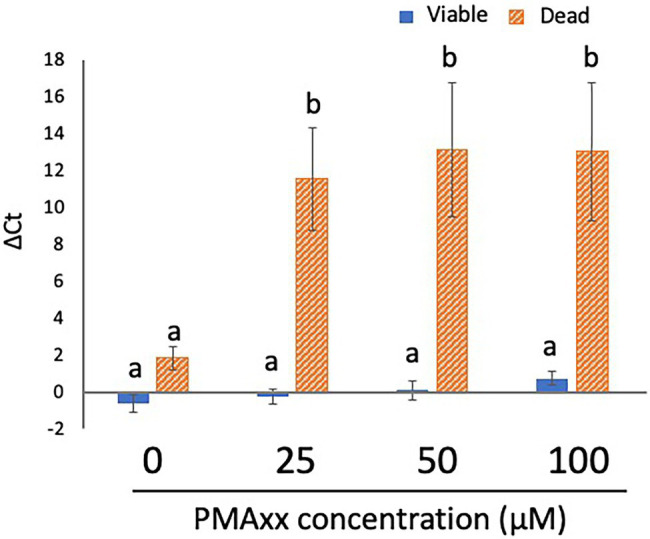
Determination of the optimal concentration of PMA. Two hundred microliters each of viable and dead cell suspensions (concentration: 5 × 10^7^ CFU/ml for both) was treated with PMA Enhancer and PMAxx at various concentrations (0, 25, 50, and 100 μM) before DNA extraction and qPCR. The columns display the mean values from five independent experiments ± standard deviation. The different letters indicate significant differences among different groups ( *p* < 0.05). PMA, propidium monoazide; CFU, colony-forming unit; and qPCR, quantitative PCR.

Although the manufacturer recommended a PMAxx concentration of 25 μM, some studies have reported that a higher concentration of PMA more strongly inhibits the qPCR signals from dead cells (Lv et al., 2019; Miotto et al., 2020). To determine the optimal concentration of PMA, 200 μl each of the viable cell and dead cell suspensions (concentration: 5 × 10^7^ CFU/ml for both) was treated with PMA Enhancer and PMAxx at various concentrations (0, 25, 50, and 100 μM) before DNA extraction and qPCR. The qPCR signals from viable cells were not inhibited at the concentrations of PMAxx assessed in this study (Figure 2). In addition, there was no difference in the inhibition of qPCR signals from dead cells when 25, 50, and 100 μM PMAxx were administered (Figure 2); therefore, 25 μM PMAxx was used for the subsequent experiments.

Next, viable and dead cells at the concentration of 5 × 10^5^ CFU/ml were treated with PMA Enhancer and 25 μM PMAxx to examine the inhibitory effect of optimized PMA treatment on dead cells. The qPCR signals were detected not only from viable cells but also from dead cells, although the Ct values of dead cells were considerably greater than those of viable cells (Supplementary Table S1). Accordingly, we performed the two-round PMA treatment, anticipating that it would exert a stronger inhibitory effect on the qPCR signals of dead cells. qPCR signals were not detected from all dead cells tested in this experiment but were detected from live cells (Supplementary Table S1). These data indicated that two-round PMA treatment completely inhibited the qPCR signals from dead cells; therefore, we performed two-round PMA treatment in the subsequent experiments.

### Detection of *Campylobacter* spp. in Spiked Chicken Juice

**Figure 3:**
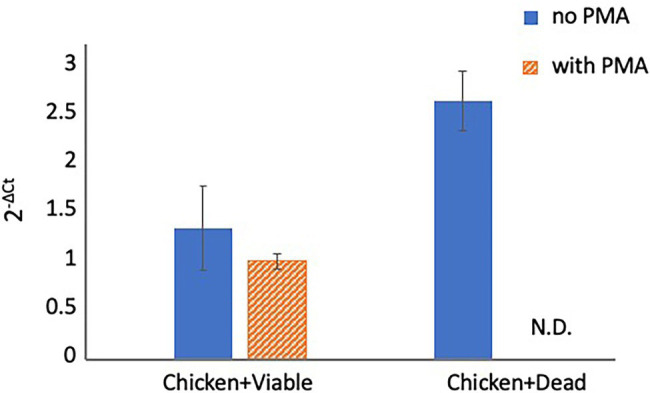
Detection of *Campylobacter* spp. in spiked chicken juice. The chicken juice was spiked with 5 × 10^4^ CFUs of viable cells and dead cells and treated with PMA before DNA extraction and qPCR. The columns display mean values from three independent experiments ± standard deviation. CFU, colony-forming unit; N.D., not detected; PMA, propidium monoazide; and qPCR, quantitative polymerase chain reaction.

To evaluate whether PMA can inhibit qPCR signals from dead cells under organic matter-enriched culture conditions, the inhibitory effect of PMA was examined in chicken juice. First, two-round PMA treatment was performed using chicken juice spiked with 200 μl of viable and dead cell suspensions (concentration: 5 × 10^4^ CFU/ml for both) before qPCR. Because the number of *Campylobacter* spp. cells naturally contaminating chicken samples is known to be relatively small (Scherer et al., 2006; Duqué et al., 2018; Ohnishi and Hara-Kudo, 2021), we used cell suspensions at this specific concentration. The results of qPCR with and without PMA treatment are shown in Figure 3. PMA did not inhibit the qPCR signals from viable cells, whereas it completely inhibited the qPCR signals from dead cells. These results indicated that the two-round PMA treatment completely blocked the qPCR signal.

### Detection of *Campylobacter* spp. in Naturally Contaminated Chicken Meat

Table 2 summarizes the results of *Campylobacter* spp. detection in 26 chicken meat samples using qPCR preceded by two-round PMA treatment or using a conventional culture method. When PMA-qPCR was used, 19 chicken meat samples tested positive for *Campylobacter* spp., whereas when the conventional culture-based method was used, 14 chicken meat samples tested positive. The detection rate for PMA-qPCR was higher than that for the culture-based method, particularly in chicken liver samples.

**Table 2:** Number of *Campylobacter* spp.-positive chicken meat samples determined using PMA-qPCR or a conventional culture-based method.

Sample type	PMA-qPCR	Culture^*^	N
Whole leg	9 (100%)	8 (88.9%)	9
Breast filet	4 (44.4%)	3 (33.3%)	9
Liver	6 (75.0%)	3 (37.5%)	8

* Detection of Campylobacter spp. was confirmed by direct sequencing using C412F and C1228R primers listed in Table 1. PMA-qPCR: propidium monoazide treatment combined with quantitative polymerase chain reaction.

## Discussion

The detection of *Campylobacter* spp. in chicken meat is important for assessing the risk of campylobacteriosis. The prevalence of *Campylobacter* spp. in chicken meat is usually monitored by conventional culture-based methods (Josefsen et al., 2015), which cannot detect VBNC bacteria. While PCR can be used to detect *Campylobacter* spp., including VBNC cells, it cannot be used to distinguish between viable and dead cells. Here, we optimized a method combining qPCR and PMA treatment to detect viable *Campylobacter* spp. cells but not dead cells and evaluated the detection rate of *Campylobacter* spp. from commercial chicken meat using PMA-qPCR.

Although studies performed using *Legionella pneumophila* and *Xylella fastidiosa* recommended the use of PMA (or PMAxx) without PMA Enhancer (Kontchou and Nocker, 2019; Sicard et al., 2019), in the present study, PMA Enhancer significantly inhibited the detection of dead cells. PMA Enhancer increased the penetration performance of PMAxx, and we recommend the use of PMAxx with PMA Enhancer. The inhibitory effects depending on the PMAxx concentrations were also examined. Our data indicated that PMAxx administered at the different concentrations tested in this study did not affect the inhibition of qPCR signals from viable or dead cells. Similar results were reported in other studies that used PMA for the detection of *Campylobacter* spp. cells (Pacholewicz et al., 2013; Lv et al., 2019). Therefore, as recommended by the manufacturer, 25 μM PMAxx is sufficient for inhibiting the detection of dead cells without reducing the viability of cells. However, treatment with the PMA Enhancer and 25 μM PMAxx did not completely inhibit the qPCR signals from dead cells. We attempted a two-round PMA treatment, which led to the complete blockade of qPCR signals from dead cells. Repeated PMA treatment was reported to improve the inhibition efficiency in a study performed using *Listeria monocytogenes* (Pan and Breidt, 2007). Previous studies concluded that PMA treatment could completely inhibit the detection of dead bacteria (Josefsen et al., 2010; Lv et al., 2019), whereas other studies have indicated the limitations of PMA treatment in the inhibition of dead bacteria detection (Pacholewicz et al., 2013; Seinige et al., 2014; Duarte et al., 2015). This might be attributed to the different treatment protocols among the laboratories (e.g., the model of thermal cycler, PCR enzyme, and PCR primers used, among other factors). Therefore, researchers should first confirm the inhibition efficiency before PMA treatment is performed to inhibit the detection of dead cells. If a low inhibition efficiency is observed, repeated PMA treatment, as performed in this study, may improve the inhibition efficiency.

Using optimized PMA treatment, we detected *Campylobacter* spp. in commercial chicken meat, and the rate of detection achieved using PMA-qPCR was slightly higher than that achieved using a conventional culture-based method. This suggests that VBNC *Campylobacter* spp. may be present in commercial chicken meat. This is the first report to demonstrate the presence of VBNC *Campylobacter* spp. in Japanese commercial chicken meat. Although two-round PMA treatment completely inhibited the qPCR signals from dead cells, we prepared the dead cells by thermal lysis. However, Vondrakova et al. (2018) reported that killing methods affect PMA-qPCR. In addition, we did not examine the PMA-qPCR using experimentally induced VBNC cells, suggesting that PMA-treated VBNC cells have less qPCR signals than culturable cells. Thus, there is a possibility that some of the signals detected in qPCR with two-round PMA treatment might come from dead cells. Viable *Campylobacter* spp., including VBNC cells, have been detected in chicken carcasses using PMA-qPCR in studies performed in different countries (Josefsen et al., 2010; Pacholewicz et al., 2013; Duarte et al., 2015; Lv et al., 2019); however, in some studies, the efficiency of PMA for inhibiting dead cell detection was not assessed, or the complete inhibition of dead cell detection was not reported (Duarte et al., 2015; Castro et al., 2018), suggesting the possibility that some detected signals may have been derived from dead cells. Only one study confirmed the inhibition of signals from dead cells and detection of only viable cells in chicken carcasses (Josefsen et al., 2010). Josefsen et al. (2010) reported that the PMA-qPCR-based cell count was lower than the culture-based cell count in some naturally infected chickens, suggesting the low accuracy of the standard curves of qPCR or overestimation of the culturable cell count. Accurate quantification using qPCR depends on the quality of the standard curve, which might be difficult to ensure, especially in the evaluation of food samples (Cankar et al., 2006; Kim et al., 2014); thus, we only performed qualitative experiments.

## Conclusion

In conclusion, we optimized the protocol for PMA treatment performed prior to qPCR for distinguishing viable cells from dead cells, and our results indicated that the two-round PMA treatment completely inhibited the qPCR signals from dead cells. Two-round PMA treatment, which is simple but effectively inhibits dead cell detection, will help address the limitations of PMA treatment in the differentiation between viable and dead cells, which have been reported previously (Pacholewicz et al., 2013; Seinige et al., 2014). Using optimized PMA-qPCR, viable *Campylobacter* spp., including VBNC cells, were successfully detected in commercial chicken meat. Further monitoring using PMA-qPCR may help determine the actual prevalence of *Campylobacter* spp., including VBNC cells, in commercial chicken meat, which will contribute to reliable risk assessment.

## Data Availability Statement

The original contributions presented in the study are included in the article/Supplementary Material, further inquiries can be directed to the corresponding author.

## Author Contributions

AO contributed to the experimental design, conducted bench work, and wrote the manuscript. MT and MR contributed by conducting bench work and editing the manuscript. YI contributed to the experimental design, manuscript writing, and editing. All authors contributed to the article and approved the submitted version.

## Funding

This work was partly supported by a Health Labour Sciences Research Grant (grant number 20KA3003) from the Ministry of Health, Labour, and Welfare, Japan. This study was also supported in part by the Grant for Joint Research Program of the Research Center for Zoonosis Control, Hokkaido University, from the Ministry of Education, Culture, Sports, Science and Technology, Japan.

## Conflict of Interest

The authors declare that the research was conducted in the absence of any commercial or financial relationships that could be construed as a potential conflict of interest.

## Publisher’s Note

All claims expressed in this article are solely those of the authors and do not necessarily represent those of their affiliated organizations, or those of the publisher, the editors and the reviewers. Any product that may be evaluated in this article, or claim that may be made by its manufacturer, is not guaranteed or endorsed by the publisher.

## Abbreviations

VBNC: viable but non-culturable
qPCR: quantitative polymerase chain reaction
PMA: propidium monoazide
PMA-qPCR: PMA treatment combined with qPCR
CFU: colony-forming unit
PBS: phosphate-buffered saline
IAC: internal amplification control
dPCR: digital polymerase chain reaction
