# Supplementary material for: Two-Round Treatment With Propidium Monoazide Completely Inhibits the Detection of Dead Campylobacter spp. Cells by Quantitative PCR
Source: Front Microbiol. 2022 Apr 25;13:801961. doi: 10.3389/fmicb.2022.801961 (PMC9082804; doi:10.3389/fmicb.2022.801961)
Supplement: Supplementary file 1 [file Data_Sheet_1.PDF]

Supplemental Table S1. Average Ct values of single- and two-round PMA[*1] treatment.

CFU[*2]/mL		10^1	10^2	10^3	10^4
Single-round	Viable	35.7	33.2	28.3	24.5
	Dead	36.9	35.4	35.8	35.1
Two-round	Viable	38.4	34.2	29.6	26.8
	Dead	ND[*3]	ND	ND	ND

[*1] PMA, propidium monoazide; [*2] CFU, colony-forming unit; [*3] ND, not detected
